# A review of the idiodynamic method as an emerging research method for the investigation of affective variables in second language acquisition

**DOI:** 10.3389/fpsyg.2022.1002611

**Published:** 2022-08-25

**Authors:** Miaoyan Lu

**Affiliations:** School of Music and Dance, Baise University, Baise, China

**Keywords:** affective variables, complex and dynamic systems theory (CDST), idiodynamic method, second language acquisition (SLA), L2 learners

## Abstract

Recent years have witnessed the influence of the complex dynamic systems theory (CDST) in the field of second language acquisition (SLA). Thus, new research methods have also been introduced to meet the requirements of investigating the dynamic nature of learner-related factors including L2 affective variables. Among the innovative quantitative research methods which is compatible with the CDST is the idiodynamic method, the application of which is on the rise in SLA research. In this paper, an overall introduction to the idiodynamic method is presented first, followed by a review of the existing literature in SLA studies. Then, it is discussed why this innovative research method is suitable to investigate the dynamic nature of L2 learners’ affective variables in the complex network of classroom learning. Also, several relevant research questions that can potentially be formulated and answered using the idiodynamic method are discussed. The paper ends with conclusive remarks on the need for more extensive use of innovative CDST-compatible research methods such as the idiodynamic method in the prospective SLA line of inquiry.

## Introduction

There are quite many research procedures that mainly deal with average scores from a number of cases (for example representing human actors). These procedures rely on a hypothetical mean score to represent the central tendency of the cases as well as variability on a group scale. This can suffice for several purposes. However, the body of research in the light of the complexity and dynamic systems theory (CDST) reaches beyond this in a search for a more profound knowledge of the mechanisms accounting for a phenomenon of interest. Perceived this way, the cases of idiosyncrasies are not seen as a mere noise to be discarded in estimating a neat single mean score. We call this the ergodicity issue. When the constituent elements of a group are very close to each other, we come up with an ergodic ensemble, in which generalization is justified from the individual to the group level. Yet, this condition is mostly lacking in data. This issue has been significantly prevalent and challenged in the body of research within the past decade (e.g., Larsen-Freeman, 2006), as pinpointed by Lowie and Verspoor (2019).

For instance, when one learner is zoomed in on by a CDST researcher, a major point to consider is the timescale of the study, which may vary from seconds to minutes or from days to months or even longer. It may become hard when it is the matter of seconds and minutes. A specific tool is needed to gather authentic, real-time evidence about this learner’s intra-personal variability throughout, for example, a particular language task that takes a couple of minutes or only seconds (see also Chinn and Sherin, 2014). That was the reason why the idiodynamic approach was originally developed (MacIntyre, 2012). It sought to unravel dynamic changes through time and the potential underlying reasons. Thus, the idiodynamic approach helps a CDST practitioner to closely focus on the phenomenon during a short scale of time, with the help of analyses that rely on the changes happening to a particular individual or case that occur through the passage of time. Besides, the idiodynamic method can be used together with other research techniques the researcher may want to use, adding a new dimension and depth to the analysis of the phenomenon of interest. Two main properties of complex systems according to CDST is their self-organization and feedback-sensitivity. Self-organization indicates that complex systems self-organize into different states across time. This self-organization occurs because complex systems are feedback sensitive and due to this sensitivity to situational factors or attractors, their developmental path might settle into different attractor states.

The idiodynamic method is rooted in the idiographic approach to research with a person-centered perspective. The dynamic turn in the field of SLA has encouraged interested researchers in the L2 affective domain to shift from a nomothetic or variable-centered approach to an idiographic one. The idiographic approach can be contrasted with the nomothetic approach. The former seeks to identify what is particular to each individual, while the latter seeks for generalized rules to be used for many individuals together. Nevertheless, as described by Rosenzweig (1986), the behavior needs to be approached from three, not two, correlative approaches: nomothetic (mental rules that can be applied to all or nearly all cases), demographic (quantitative generalizations from a specific group of cases) and idiodynamic. The idiodynamic approach deals with the indicators of an idioverse, or a set of conditions phenomenologically experienced by a specific person (Rosenzweig and Hackney, 2004).

## Distinctive features of the idiodynamic approach

One main theoretical support for the use of the ididoynamic method in the investigation of L2 affective variables is the fact that language learners are not ergodic ensembles (see Lowie and Verspoor, 2019). That is, the data of individual language learners cannot be generalized to the group of these learners. Thus, the idiodynamic method provides deep insights into the dynamic development of L2 affective variables of each language learners. The collected data via the idiodynamic method is unique to each individual and might not overlap with those of the other individuals. An idiodynamic method has certain advantages (Velicer et al., 2014). To begin with, the procedures of collecting and analyzing the data are focused on intra-personal variation which makes it possible to use a variety of data to explore new research questions and can reveal interesting dimensions of those data. Secondly, this method can be used in contexts in which older interpersonal research designs might be not suitable or hard to apply. Thirdly, the idiodynamic approach has significant benefits to the investigation of patterns of change from time to time, and may be employed to indicate how certain behavior is developed and can explore the interaction among variables through time. Also, given the situated and ecologically emic and subjective perspective of the idiodynamic method with a person-centered focus on psychological constructs including L2 affective variables, the idiodynamic method holds a microscope perspective toward the states of change and stability regarding these constructs. However, it can be embedded in large scale dynamic designs with an integration of several time-scales at the same time. For instance, the idiodynamic approach can be embedded in a time-based scheme of ecological momentary assessment on a given L2 affective variable, such as anxiety or boredom, with a focus on different time scales from micro to macro perspectives. It should be noted that besides being an asset for the exploration of dynamics of L2 affective variables, the method has been revealed to contribute to the conceptualization of new constructs in this domain. For instance, Talebzadeh et al. (2020) via the use of the idiodynamic method in their study could provide evidence for the concept of foreign language enjoyment contagion. Finally, the software programs developed for the collection of the idiodynamic data contributes to both a systematic and transparent data collection and data analysis procedure. It should be noted that the details delineated for the procedures of data collection and data analysis of the idiodynamic data should be followed very meticulously and systematically to provide transparent and replicable details of these procedures. Given the exploratory nature of the idiodynamic method and the need for transparency and systematicity in both the data collection and data analysis, both of these procedures can be merged. Thus, the procedure of data collection continues until the saturation level is met and the most suitable case for the interpretation of the collected idiodynamic data is selected.

These points need to be elaborated on more in here. The idiodynamic method takes advantage of procedures to scrutinize moment-by-moment (or minute-by-minute or the like) changes within one person or case, and allows for seeking the answer to useful questions. Here are some instances within the SLA domain:

• How can self-assessed learner-related variables (e.g., motivation, anxiety, boredom, foreign language enjoyment) fluctuate through the developmental procedure of a task?
• How can self-assessed variables relate to assessments made by for example a researcher, a peer or a specialized observer?
• What are these fluctuations or changes attributed to?
• What other variables (e.g., language learners’ level of proficiency, interlocutors) affect self-assessments and attitudes?
• Is the kind of stimuli (e.g., audio or video only, or they both together) influential in the language learner’s self-assessments or attitudes? If so, in what tasks or activities?
• How does the training affect self-assessments and perceptions (e.g., pragmatic teaching of strategies)?
• What is the relationship between self-assessments or perceptions and several cognitive or affective constructs (e.g., passion for learning, grit, well-being, etc.)?

Evidently, research questions that can be explored using the idiodynamic approach can be observational or experimental. Contrary to the former, in the latter, purposefully specific factors are manipulated purposefully by the researcher, including the interlocutor’s current condition and the task difficulty, to assess their impact on various outcomes which can vary in type. The psychological, either cognitive or affective. Examples can include willingness to communicate, anxiety and motivation. Also, they may be linguistic, for example, morphological combinations, grammatical structures and accuracy or fluency in the language. Therefore, the idiodynamic approach helps to provide useful real-time data at the individual level for researchers and is capable of exploring new research questions to provide significant insights.

In practice, the idiodynamic approach entails recording a video of the individual (teacher or learner or whole class) and then watching the video together with the participants to do the assessments of their behavior solicited with themselves. The distinctive feature of the idiodynamic approach is that it gives the individual the chance to systematically assess every moment of the task via a special software (i.e., the user-friendly and free-of-charge Anion Variable Tester). More precisely, the suggested stages of some idiodynamic research include the individual’s engagement in a task the whole procedure of which is videotaped, loading the video soon afterward on the above-mentioned software, then asking the participant to view the video and make an assessment of a particular variable (e.g., passion for learning, anxiety, enjoyment, and boredom) in each moment of the videoed episode (which entails quantitative data), asking the individual (through stimulated recalls) (Gass and Mackey, 2000) to explain his/her assessments (which involves qualitative data). Then, a graph is produced by the software along with an Excel file including the assessments while the researcher can transcribe the qualitative data for more analytical phases. The researcher can do a horizontal analysis depending on the objective of the study, and compare patterns among different individuals. Also, the researcher can do a vertical analysis to focus on intra-personal patterns.

Idiodynamic approach with its focus on variability and temporal changes helps to provide unique data which are compatible with CDST. There is no entire reliance on memory in this method as a recorded video is used which is a cue to the individual to elicit data. There is, however, the possibility of biased reports in self-assessments. The generated data can be large in size and might also seem messy, particularly when many participants are involved. Also, the data can be largely idiosyncratic and situated. Thus, the researcher should remember that s/he is supposed to somehow associate the findings to the existing theories or practice. It should be noted that the emic subjective nature of the idiodynamic method does not limit its scope in terms of the variables to be targeted for exploration at the same time. This means that more than one affective variable can be explored simultaneously. For instance, the dynamics of both enjoyment and anxiety can be the focus of an idiodynamic study.

What follows is a review of the literature in L2 studies and more specifically on L2 affective factors that have employed the idiodynamic approach in research methodology. The findings and implications for the field will show why it is worth waiting to see many more academic publications to report the findings of quantitative studies using the idiodynamic method.

## Review of idiodynamic method in L2 affective factors

The studies in L2 education domain which have used the idiodynamic method to explore language learners’ affective variables are still limited in number, and they have been all conducted within the past few years. Yet, they had a significant contribution to the field, as will be reviewed here. In advance to the review, we should like to draw attention to the significance of L2-related affective factors. Mind that the existing body of research in SLA so far has shed light on multiple affective variables which can be involved in L2 learning, such as L2 learners’ self-confidence (de Saint Léger and Storch, 2009; Peng and Woodrow, 2010; Lee and Lee, 2019), L2 anxiety (MacIntyre and Legatto, 2011; Lee, 2019; Lee and Lee, 2019), different types of language learning motivation (MacIntyre et al., 2002; Yu, 2011; Khajavy et al., 2016; Lee, 2019; Lee and Lee, 2019; Pishghadam et al., 2021), grit (Duckworth et al., 2007; Lee and Lee, 2019), as well as boredom (Derakhshan et al., 2021; Pawlak et al., 2021). Though these affective variables have long been of interest to SLA researchers, studies enlightened by the CDST are still limited. As described by Dewaele and Li (2020), SLA has entered the third phase (i.e., the dynamic phase) of investigating affective variables, following the general and domain-specific phases. This dynamic turn is marked by a concern for both positive affective variables (e.g., motivation, self-confidence, grit, and foreign language enjoyment) and negative constructs (e.g., demotivation, anxiety, and boredom). There has been a shift in SLA research to examine the development of learner-related affective variables by tracing their complex dynamic interactions. A summary of these published works of research are included in Table 1.

**TABLE 1:** A summary of the literature of the idiodynamic method and L2 affective variables.

Title of paper	Authors (year)	Journal	Purpose of study	Main findings
A dynamic system approach to willingness to communicate: developing an idiodynamic method to capture rapidly changing affect	MacIntyre and Legatto (2011)	Applied Linguistics	To study variations in willingness to communicate using an idiodynamic method	The research showed consistency and variation both in willingness to communicate in a relatively homogeneous group of speakers.
The idiodynamic Method: A closer look at the dynamics of communication traits	MacIntyre (2012)	Communication Research Reports	To explore the affective or cognitive features that emerge out of human interactions	They proved the effectiveness of idiodynamic method as an innovative method to explore the quick changes in dynamic systems that account for the process of communication
The motion of emotion: idiodynamic case studies of learners’ foreign language anxiety	Gregersen et al. (2014)	The Modern Language Journal	To assess language learners’ anxiety, its causes, and the interpretations of rapidly changing affective reactions during a short time	A strong association was found between the different sources of converging data and proved the strength of taking into account L2 learners on a personal level using triangulated quantitative and qualitative methods.
English as a foreign language learners’ anxiety and interlocutors’ status and familiarity: An idiodynamic perspective	Elahi Shirvan and Talebzadeh (2017)	Polish Psychological Bulletin	To explore the dynamics of anxiety EFL learners experience, especially during the moments of interacting through conversations	The results showed both variation and stability in the L2 learners’ anxiety influenced by the speakers’ acquaintance with the participants and their condition and also their verbal and non-verbal feedback.
Exploring the fluctuations of foreign language enjoyment in conversation: an idiodynamic perspective	Elahi Shirvan and Talebzadeh (2018a)	Journal of Intercultural Communication Research	To explore the effect of different topics on the dynamics of a main variable in positive psychology: foreign language enjoyment	The results showed the dynamicity of enjoyment emerged not only intra-individually, but also inter-individually influenced by conversational topics
Is transparency an illusion? an idiodynamic assessment of teacher and peers’ reading of non-verbal communication cues of foreign language enjoyment	Elahi Shirvan and Talebzadeh (2018b)	Journal of Intercultural Communication Research	To explore the extent to which experiencing enjoyment in communicating in EFL is clear to the teacher and peers inside the ecology of classroom learning	The results showed mixed and dynamic outcomes which proved the unstable and complex quality of enjoyment and showed that transparency of FLE in communication is an illusion.
Enjoyment and anxiety in second language communication: An idiodynamic approach	Boudreau et al. (2018)	Studies in Second Language Learning and Teaching	To test the association between anxiety and enjoyment using an idiodynamic method	They found that the association between enjoyment and anxiety is significantly dynamic, which leads to different patterns of correlation either negative or positive.
Rapid changes in foreign language learning anxiety caused by a multiplicity of topics: an idiodynamic approach	Saghafi and Elahi Shirvan (2020)	Journal of Language & Education	To investigate different topic-based kinds of FLA	The main factors influencing the FLA dynamics were linguistic block, topic familiarity, topic interest.
The idiodynamic method: Willingness to communicate and anxiety processes interacting in real time	MacIntyre and Gregersen (2022)	International Review of Applied Linguistics in Language Teaching	To exemplify the usefulness of idiodynamic method in exploring L2 related variables	The strengths and limitations of the method were shown within an example study in the assessment of WTC and CA.
The idiodynamic method: A practical guide for researchers	MacIntyre and Ducker (2022)	Research methods in Applied Linguistics	To introduce the features and procedures of the idiodynamic approach	The distinctive features and step-wise procedures were clarified and exemplified.

A pioneering research in SLA studies that used the innovative idiodynamic methodology was conducted by MacIntyre and Legatto (2011) to trace the fast changes in language learners’ willingness to participate. The research procedure involved recording responses from a sample of young female adult speakers to L2 communicative activities, their self-assessments of changes in willingness to communicate during those activities, and reporting their experience and attributions for variations in the variable of interest. The role of speakers’ fixed personality traits was considered along with the observations offered during the participants’ speech by an observer. The authors pinpointed that viewing willingness to communicate as a dynamic system helps to examine the changes in this variable through time. The analysis was indicative of consistency and variation both in willingness to communicate even among an almost homogeneous group of L2 speakers. They found that searching memory for vocabulary was a major process that affected willingness to communicate, though arguably other variables (such as L2 anxiety) can also influence the willingness to communicate. The researchers concluded that willingness to communicate can be viewed a dynamic system and made suggestions on how to deal with the limitations of the idiodynamic approach in future studies.

A year later, MacIntyre (2012) referred to the idiodynamic method as a new way of studying the affective or cognitive factors involved in human communication. They elaborated on the procedures included within a study following a idiodynamic approach. They also exemplified L2-related factors that can best be explored using this innovative research method. These included the self-ratings of perceived competence, willingness to communicate, and communication apprehension. MacIntyre (2012) contended that this method made it possible to examine the individual’s different interconnected dynamic systems in real time.

In a similar line of research, Gregersen et al. (2014), firstly drew attention to the dynamic nature of language learning as an emotionally and psychologically mechanism influenced by many ever-changing variables and emotional traits that cause momentary changes in students’ adaptation skills. In this study, Gregersen et al. (2014) triangulated physiological, idiodynamic, interview, and a survey data of both high- and low-anxiety L2 learners to assess their language anxiety, its causes, and the explanations of quickly changing affective states during a short period of time. The L2 learners’ presentations were videotaped using heart monitors, in their EFL class. Then the idiodynamic method was used for the participants to self-rate the anxiety they felt every moment for 3.5 min and then, in an interview, they elaborated on their responses. The strong correlation found among the different sources of data proved the significance of investigating language learners on a personal scale via mixed quantitative and qualitative research methods. The study had pedagogical implications for how to explore both positive and negative emotions, facilitate the reinterpretation of physiological cues, plan ways to help students stay active in communication tasks, and taking the best advantage of the positive power of pre-planning, planning, and practicing.

In another study, Elahi Shirvan and Talebzadeh (2017) explored L2 anxiety in SLA. They drew attention to the dynamics shift in the SLA research domain, and the need for unravelling the dynamics of L2 anxiety, particularly during the moment-by-moment interactions during conversations. Elahi Shirvan and Talebzadeh (2017) contended that the dynamics of anxiety may appear in different forms out of these interactions, influenced by the network of the individuals’ interlocutors and their familiarity with these interactions. They followed an idiodynamic approach to the examination of the dynamics of L2 learners’ anxiety during their interactions with different interlocutors. In this case study, the participants were two female freshmen in a speaking and listening class at university. They were interviewed by four interlocutors of a different level and familiarity level. Then, these participants self-assessed the fluctuations in their anxiety influenced by each interlocutor taking part in each conversation. Next, stimulated recall interviews were used to unravel the causes of variation in the students’ anxiety provoked within the conversations. The Findings revealed both stability and change in the students’ anxiety influenced by the interlocutors’ familiarity with the participants, their level and also the kind of verbal or non-verbal feedback involved. These changes were explained based on the major features of CDST.

In a similar work of research, Elahi Shirvan and Talebzadeh (2018a) attempted to explore the effect of a number of topics on the dynamics of foreign language enjoyment as a main construct in positive psychology. They employed an idiodynamic approach to trace the fast momentary variations in FLE. To this aim, they recruited a small sample of female university students involved in a conversation with simple and difficult topics both, and self-assessed their momentary experience of enjoyment during the process of conversation. The findings showed that the dynamicity of enjoyment appears not just intra-individuals, but inter-individually as well depending on the topics of conversation. Also, the idiodynamic method gave us the chance to explore the latent factors that account for the dynamic changes to foreign language enjoyment and provide a better insight into FLE as a dynamic system.

In the same year, the same researchers focused on the emergence of both positive and negative emotions in three groups of six university students engaged in a TV program which criticized English-speaking literature as an imagined community. The program included setting a particular day to discuss a story and promising to publish the best final report in a credible literary journal. Each community member formed a particular imagined identity in each group as a critic, photographer, reporter, and presenter all competing together for a final nomination of the best critique and presentation on the final day and also publishing the best report. The triangulated data were analyzed qualitatively. Elahi Shirvan and Talebzadeh (2018b) concluded that the emergence of enjoyment resulted from the imagined identities formed in the classroom as well as facilitative negative emotions including anxiety which led to the establishment of the individuals’ affiliations imagined.

The idiodynamic approach was also used by Boudreau et al. (2018) to explore the relationship between anxiety and enjoyment. These researchers contended that the idiodynamic approach is best suited for exploring affect, and in particular discrete emotions such as L2 anxiety, because these emotional phenomena are highly dynamic and situated. Boudreau et al. (2018) included 10 English native speakers who were learning French as their L2. They asked the participants to carry with them a photo of an object they perceived as joyous to the laboratory. Upon arriving in the lab, the participants were given 2 tasks to do: a photo narrative and an oral interview task (in a counterbalanced order). In the former, the participant would explain the photo in the L2 and why s/he found it enjoyable. In the latter, a number of questions were asked from the participant with a rising level of difficulty. Both tasks were video-recorded and immediately analyzed. The videos were then played for the participant for a self-assessment of anxiety and enjoyment independently (with a counterbalanced order). The participants needed to do their assessment of anxiety/enjoyment say to what they attributed the stability or changes in these assessments.

In their quantitative data analysis, for each participant, Boudreau et al. (2018) examined the correlations between anxiety and enjoyment. They found a negative relationship in general that ranged from medium to strong. Yet, when they analyzed it more deeply, they found that this pattern was far from consistent. Therefore, the researchers suggested the further use of idiodynamic studies in natural learning environments including the classroom. However, the researchers also highlighted the significance of immediate assessments and stimulated recalls to benefit from the respondents’ fresh memory.

More recently, Saghafi and Elahi Shirvan (2020), acknowledged the dynamic quality of L2 anxiety. They used an idiodynamic procedure in exploring topic-based kinds of L2 anxiety. In advance to the study, a total number of 20 female intermediate EFL learners were recruited using the L2 learner anxiety scale. To this aim, two groups of the same size were included, one with low-anxiety learners and the other with high-anxiety learners. They video-recorded the students’ answers to four questions which were topic-based. The students were asked to self-assess the ups and downs of L2 anxiety meanwhile they were answering the probes. They helped to find causal traces of the topic-based fluctuations in L2 anxiety. The results revealed both intra-individual and inter-individual variation and stability in L2 anxiety. The main factors that affected the dynamics of L2 anxiety were linguistic block, familiarity with topic, interest in topic, and the emotional loading related to the topic.

Along the same line of inquiry, MacIntyre and Gregersen (2022) drew attention to the usefulness of the idiodynamic approach in studying the real time complex dynamics of mixed affective and cognitive phenomena which constantly interact with human communication. These researchers reviewed the procedural steps involved in the idiodynamic approach and pinpointed that the idiodynamic method can be used to examine language learning-related factors such as motivation, perceived competence, willingness to communicate, self-efficacy, empathy, strategy use and similar variables. MacIntyre and Gregersen (2022) reviewed the strengths and limitations of the idiodynamic method and exemplified its use in testing willingness to communicate and L2 anxiety.

Finally, as the latest work of research on idiodynamic method and L2 affective variables, MacIntyre and Ducker (2022) provided a practical guide to the use of idiodynamic method in SLA research. These researchers explained why this method was strongly needed in SLA research to provide a more holistic image of the inherent complexity of language learners individually. MacIntyre and Ducker (2022) emphasized on the advantages of using the idiodynamic approach to explore language learners’ emotions and provided some examples from the existing literature to further elucidate the procedures involved.

## Directions for future research

The use of the idiodynamic method in the recent studies of the L2 affective domain have provided stronger evidence for stative nature of some L2 affective variables such as enjoyment and anxiety in the process of foreign or second language learning. This means that these emotions susceptible to momentary factors go through change. Thus, this espoused dynamic perspective to such emotions via the use of the idiodynamic method can also provide language teachers with this dynamic mindset toward these emotions in their interactions with their language learners within the environment of L2 classroom. Considering the increasing line of research with a dynamic approach, there is a considerable need for methods of estimation that manage to consider intra-individual variations and inter-individual differences. The idiodynamic approach used in the above-mentioned studies enabled the researchers to focus on within-individual changes as they actually occurred moment by moment in the real time of classroom learning. This method results in more accurate and nuanced conclusions concerning the L2 learner related affective factors. The main challenge researchers interested in the use of the idioynamic method might face in their exploration of the L2 affective variables via the method might be related to the procedures of data collection and data analysis, which need to be organized very systematically. This systematicity contributes to the trustworthiness, replicability, and transparency criteria for the evaluation of qualitative criteria.

The future line of L2 education research needs to essentially explore L2 learner related variables longitudinally to reflect a more realistic, dynamic conceptualization of the variable of interest as it grows naturally in the fully interactive classroom context (see Shao and Parkinson, 2021). Accordingly, it is expected that the measurement represents this dynamic shift. The quantitative data analysis procedures adopted should represent the nuanced development of the affective variable of interest and take into account the moment by moment variations and the underlying causes to those changes (in the variable). It is supposed to consider intra- and inter-individual differences among learners. Traditional statistical procedures are incapable of meeting these requirements and, therefore, only give us a static image of the variable of interest, which fails to represent its growing quality in the live context of classroom learning. Conventional research methods often take mean group scores to represent subjects’ achievement or a specific learner-related affective variable, and lose sight of intra- and inter-individual differences that naturally characterize the growth of human qualities or behaviors.

The idiodynamic approach employed in the aforementioned studies can be used as efficiently to assess other learner related factors in language learners’ (or possibly teachers) psychology such as well-being, compassion, mindful attention/awareness, loneliness and so on. In the dynamic context of a language classroom, characterized by the interactive nature of a multitude of personal (teacher or learner) and contextual variables, the idiodynamic approach can be effectively used in tracing the development of the variables in the time-dependent phases of the L2 program nested within the dynamic context of classroom learning. Idiodynamic method seems to meet the requirements of measuring multifaceted human traits from a CDST perspective and can, therefore, adequately contribute to the existing related literature in SLA domain, with a long-held interest in L2 learners’ affective variables including anxiety, enjoyment, boredom, passion for learning and so on. Moreover, the tools used in the idiodynamic method for the investigation of L2 affective variables can be expanded to the use of the physiological devices. For instance, Gregersen et al. (2014) in their investigation of the fluctuations of language anxiety used heart monitors to provide more credible evidence regarding these fluctuations. Finally, the application of the ididoynamic data in the L2 affective domain can be expanded from individual data to dyadic data in which the dynamics of L2 affective variables in the individuals involved in the dyadic interactions can be measured at the same time.

## Conclusion

In this review, awareness was raised of the failure of traditional analytic frameworks to delve into multi-faceted language learners’ affective variables such as anxiety, enjoyment, boredom, and the like. This study also emphasized the need to avoid a simplistic integrative or holistic attitude to any language learner-related affective variable and, instead, highlighted the need to trace the moment by moment trajectory of changes in language learners’ emotions, and account for intra- and inter-individual differences. The present study drew attention to the insufficiencies of the mere reliance on single-shot designs of study and the unrealistic static image of L2 learners’ emotions which can result in inefficient decision-making about individual language learners in foreign language education programs. The idiodynamic approach allows for drawing more realistic conclusions from applied linguists’ data. Idiodynamic method is capable of modelling a unique trajectory for each subject, a mean trajectory of all subjects, along with the variability of this mean (Hiver and Al-Hoorie, 2019). As the studies reviewed in this paper showed, there is still a dearth of research using the idiodynamic method in exploring language learners’ emotions. Many L2 learner or teacher-related affective variables are left unattended from a dynamic approach. Appropriate quantitative and qualitative research methods are, thus, deemed essential to embrace the complex city and dynamicity involved in the language learning process. Thus, idiodynamic research method is hoped to enrich the quantitative (or mixed-design) studies of the affective variables involved in the process of language learning.

## Ethics statement

The studies involving human participants were reviewed and approved by Baise University Academic Ethics Committee. The patients/participants provided their written informed consent to participate in this study.

## Author contributions

The author confirms being the sole contributor of this work and has approved it for publication.
